# On the Usefulness of the Medical Profession, beyond the Limits of the Profession

**Published:** 1842-12-17

**Authors:** 


### BIBLIOGRAPHICAL NOTICE.

*On the Usefulness of the Medical Profession, beyond the limits of the Profession. A Lecture Introductory to the Course of Practice of Medicine in Jefferson Medical College of Philadelphia.* Delivered November 4, 1842. By J. K. Mitchell, A.M. M.D. Published by the Class. Philadelphia: 8vo. pp. 30.

Professor Mitchell justly remarks, that "of all the professions and pursuits of life, that of medicine demands, in its ordinary preparation, the greatest extent and variety of knowledge," and hence the necessity of an extended preliminary education for those destined to the medical profession.

"The world at large associates with a defective education the idea of professional ignorance and incapacity; that many very valuable works are published in foreign languages, and that, independently of the actual acquirements in the course of a scholastic education, the habits of attention, of arrangement, and digestion, are fertile means for medical attainments. Few thinking men, therefore, such as govern public sentiment, are willing to believe that an uneducated man can acquire or does possess as extensive or as well defined a knowledge of his profession, as one who has had the gain and the discipline of scholastic instruction. For these reasons, physicians, even in this go-ahead and hasty country, are generally much better educated than the mass of beings around them, and, if we take *science*, as well as learning, into the account, than the members of the other learned professions."

This fact is abundantly shown by the numerous and daily contributions to the literature of our country by physicians of high professional rank. To the catalogue which the professor subjoins might be added his own name; for amidst the occupations and distractions of a large practice and onerous professorship, he still finds time

"To pour the unpremeditated lay."

"Dr. Comstock writes on, and teaches admirably, elocution; Dr. Bird stands in the front rank of American dramatists and novelists; Dr. Standbridge composes useful works on the philosophy and practice of music; Dr. Holmes soothes his wearied professional spirit with tones of the poet's lyre, such as command the listening ear of the country, and are heard beyond the broad Atlantic; Dr. Dunglison delivers to immense and delighted audiences, lectures on the instincts of animals; or instructs and amuses them with disquisitions on the curiosities of popular superstitions; Dr. Wood retires from his beautiful botanical collection, to pen an able essay on "The British Possessions in India." Some of you may have heard our colleague, Dr. Meigs, busy almost to impossibility, lecturing at the Athenian Institute, on the "Age of Augustus," and the "Siege of Jerusalem," with an eloquence and interest, as though nothing else could have occupied his mind for years. Dr. Morris, whose busy life every one knows, finds time to make an excellent abridgement of the Life of Wilberforce. Dr. Benjamin Coates invents a matchless balance with one hand, while he fashions with the other a racy ode to an "Antediluvian Fossil;" and his talented brother, Dr. Reynell Coates, is just offering to the world the fruits of his extensive learning in a new journal, to be called the Literary Age. Dr. Patterson, the accomplished President of the U. S. Mint, is ever ready to disburden, for public good, his powerful mind, richly stored with the wonders of Astronomy and the utilities of Natural Philosophy; while Dr. Hare, of world-renown in science applied to medicine, amuses himself and instructs others, with profound memoirs on atmospheric storms, and public currency and credit. Professor Dickson has just reached his southern home, after having delivered at Yale, the annual oration to its Alumni; a discourse of which Fame has spoken to the country. My indefatigable friend, Dr. Bell, issues in rapid succession, valuable works on baths, on health and beauty, and on the art of prolonging life; works presenting discursive knowledge of great extent and variety. Still farther from the beaten path of medicine, he wrote the lives of distinguished painters, and delivered discourses on their favourite subject to the artists of the Academy. Dr. Emerson, advancing from curious and elaborate statistics, just within the circle of medical philosophy, instructs and gratifies the class of the Franklin Institute with learned and ingenious lectures on meteorology. It is but a few days since I was absorbed in the pages of an able and interesting work, the Life of Bainbridge, the naval hero, written by Dr. Thomas Harris. If we did not now know how much has been done by the profession, for extrinsic departments of learning, we should be at a loss to conceive where the busy surgeon found time to read so much, and to write so well. Who now thinks of a voyage round the world, without recalling to mind the rich and instructive volumes of Dr. Ruschenberger! The same gentleman stoops forom the lofty field of general literature, to perform, aye, and well too, the benevolent and useful duty of forming books for schools, where the sciences are made familiar and agreeable to the young mind of the nation.

But I fear I grow tedious, not because of the worthlessness, but the very richness and prolongation of the catalogue: I will, therefore, merely allude to the travels of Mott and Gibson, the reviews of Elwyn, the musical essays of La Roche, the Siamese memoir of J. Rhea Barton, and the literary lectures of Pancoast, and Mütter, and Jackson and Condie.

I should fail in justice to the man, and in respect for the preceptor, if I did not say something of the five large volumes of "Select Speeches, Forensic and Parlimentary," collected, arranged, and published in early life, by Dr. Chapman, the taste in the selection of which, is surpassed only by the exquisite general preface, and the pertinent, elegant, and impressive remarks, with which the editor introduces each of the many orations of the extensive collection.

Is not this a wondrous view of the learning, industry, public spirit, and talent of the profession, and does it not more than excuse the enthusiastic sage of Cos, when he declares, that "whatever really constitutes wisdom is to be found in medicine?"—*(Hippoc.)*

Not alone as writers on topics either remote from, or slightly connected with, professional pursuits, do physicians stand, as a class, distinguished, at least, in our country. They are also often conspicuous for varied accomplishments, by which they not only adorn, but serve society. There is near me a surgeon, of the highest rank in his profession, who is a good modeller, an inventive designer, a man of taste, and a promoter of everything tending to elevate and refine society. I see another, who has, perhaps, in the world no superior in his art, who draws beautifully, models exquisitely, and is a master in music. Here is a botanist, there a geologist, a conchologist, a chemist, a meteorologist, a metaphysician, a politico-economist, an astronomer. The room in which I lecture, accidentally contains many professional gentlemen, each distinguished for knowledge of some one or other of the departments of general knowledge, or adorned by literary taste, or conspicuous for social accomplishments.

It is, gentlemen, when taking such a survey of the merits of the profession, that we must feel the greatest pride in it, and felicitate ourselves on being of a corps professionally so indispensable, scientifically so important, and socially so valuable to the country.

The following paragraphs contain much practical good sense, clearly and neatly expressed:

Now, it is a curious fact that among the trades, occupations and professions, medicine alone sends its votaries annually to the great fountain of metropolitan knowledge. Like as to a vast heart, the channels of public circulation bring, every successive year, from every quarter of our proudly-spread country, streams of medical aspirants; who return again a rich freight of intellectual treasure, to the unforgotten fields of kindred and friends. This mighty moral and intellectual circulation is among the richest products of the land. I waive the advantage to those who come and go, vast as it is. I overlook, for the present, the proud store of professional good poured into the recesses of the forest, carried to the sources of the distant rivers, and laid at the majestic feet of the spinal mountains of the continent. I will not even insist on the civilizing effect of a never failing contact with the great centre of refinement. But I will declare that the political value of this circulation is far above them all. It may at first view seem rather startling to say so; but let us go into the account, and, as the merchants say, strike a balance.

It cannot be denied that our happy country is tied together solely by the *force of opinion*. We have here, thank God, no other fetters than the golden chains of patriotic fellowship. It is because the men of the north, and of the south, and of the west, agree to look upon each other as countrymen, that we are an undivided people, though spread over an almost measureless territory, and inhabiting climates of the greatest dissimilarity. The hewer of granite in New Hampshire, the cultivator of wheat in Pennsylvania, the raiser of hemp in Kentucky, and the planter of cotton and sugar in Louisiana, claim as a common right, the principles of the Declaration of Independence, and look proudly up to the stars and stripes which waved at Yorktown.

The stranger who traverses our vast country, is forcibly struck with the great uniformity of our manners and language. In England the people of adjacent counties scarcely understand each other, though professing alike to speak the English language. The field labourer of Lancashire has a dialect which confounds the agriculturist of Yorkshire; either would be puzzled to translate into common English, the peculiarities of the man of Somersetshire.

It is so, also, in France, where the lower orders, as they there choose to call the peasantry and the artisans, speak such a diverse *patois*, in different districts, as to confound the ear of a Parisian. Nothing, therefore, more surprises the traveller, than the absence of a marked or unintelligible *patois* in any part of our country. It is true that we have German settlements, where they speak Dutch, French settlements, where the oral language is French; and Irish and Scotch districts, where those who relish a rich brogue, or a Doric dialect, may be thoroughly indulged. But English, when spoken by Americans, is always intelligible to Englishmen, whether they hear it in Boston or Savannah, in New York or Natchitoches. While much of this uniformity depends on the fact, that the early settlers in our country were educated men, and men who strove hard to give to their children the precious boon of scholastic instruction, yet, that our English should have stood so long pure against the corrupting influence of example imported from all nations, and of time and carelessness on our own part, can only be explained by supposing that the resort of students of all kinds, but especially of medical students, to the great common centres of learning, has in no small degree contributed to a result otherwise totally unintelligible. In the same way we may explain the unusual uniformity of manners and habits, so often commented on by foreigners, favourably or unfavourably, according to their taste or caprice.

Now I hold that of all the bonds of union by which a people, so diffusely spread as we are, can be kept united, none is more potent than the sameness [identity ?] of language and manners. It makes intercourse easy and agreeable, it promotes good fellowship, and it creates an almost instinctive horror of disseverance. The disruption would be as of one family, whose taste and sentiment, and language and manners are in kindly unison.

Of all men the physician enjoys the best opportunity of disseminating his sentiments, and conveying his example. He visits every family. He converses with the women and children, and domestics; and the more wild and remote his field of labour, the more frequently and the longer is he thrown into their society. He is not confined, like the lawyer to his court, or to contact with men, or like the clergyman, to the pulpit or the rare visitation. He is often almost domesticated; he is looked up to confidingly, and he is considered as competent authority in most of the things that usually interest a domestic circle. His opportunity, therefore, of impressing his manners and habits and language on the people is unparralleled.

Stored with science, and learning accumulated both at classical seminaries and schools of medicine, the educated physician is usually disposed to contribute a portion of his knowledge to the benefit of his own little community. He therefore is, both from capacity and disposition, foremost in promoting the literary enterprize of his district. How few lyceums are built up or carried forward without his agency. Few weeks pass without an application to me to deliver a lecture in some near or distant lyceum; and it rarely happens that the invitation is not brought or sent by some liberal physician, who not only teaches himself, but solicits aid from others. Giving as much credit to other professions as is their due, we must admit that in this, as in a thousand ways, the American faculty of medicine, by making uniform our manners, enlightening our sentiments, and invigorating our reason, adds immensely to public security and public union.

